# Seed longevity and genome damage

**DOI:** 10.1042/BSR20230809

**Published:** 2024-02-28

**Authors:** Wanda Waterworth, Atheer Balobaid, Chris West

**Affiliations:** Centre for Plant Sciences, University of Leeds, Woodhouse Lane, Leeds LS2 9JT, U.K.

**Keywords:** DNA repair, genome stability, germination, mutation, recombination, Seed

## Abstract

Seeds are the mode of propagation for most plant species and form the basis of both agriculture and ecosystems. Desiccation tolerant seeds, representative of most crop species, can survive maturation drying to become metabolically quiescent. The desiccated state prolongs embryo viability and provides protection from adverse environmental conditions, including seasonal periods of drought and freezing often encountered in temperate regions. However, the capacity of the seed to germinate declines over time and culminates in the loss of seed viability. The relationship between environmental conditions (temperature and humidity) and the rate of seed deterioration (ageing) is well defined, but less is known about the biochemical and genetic factors that determine seed longevity. This review will highlight recent advances in our knowledge that provide insight into the cellular stresses and protective mechanisms that promote seed survival, with a focus on the roles of DNA repair and response mechanisms. Collectively, these pathways function to maintain the germination potential of seeds. Understanding the molecular basis of seed longevity provides important new genetic targets for the production of crops with enhanced resilience to changing climates and knowledge important for the preservation of plant germplasm in seedbanks.

## Introduction

**Figure 1:**
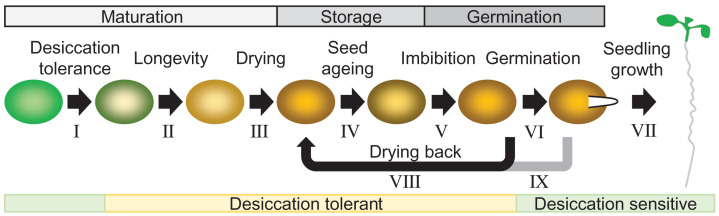
Critical stages in the life of a seed The key stages from seed maturation to seedling establishment have been the subject of recent reviews: the acquisition of desiccation tolerance (I) is followed by a developmental programme that extends longevity during storage (II) [13]. Maturation drying (III) decreases seed moisture content to ∼10% fresh weight and solidifies the cytoplasm into an intracellular glass [3,13]. Storage of seeds under ideal conditions of low temperature and low humidity extends survival whereas suboptimal environmental conditions result in seed ageing (IV) [3,30,32,150]. Imbibition (water uptake) initiates metabolism and cellular repair (V) which is followed by germination (VI) in non-dormant seeds [14,151,152]. The impact of seed ageing extends into post-germinative growth (VII) [29,122]. Seed imbibition is reversible (VIII): seeds in the soil undergo hydration-desiccation cycles. Commercial seed priming technologies hydrate seeds, followed by a dry back, to facilitate cellular repair and improve the vigour of germination and seedling growth [128,129]. Desiccation tolerance is lost as seeds progress to germination but can be re-established by treatment with ABA or PEG (IX) allowing survival after re-drying [13].

The ability of plants to produce desiccation tolerant seeds provides a highly successful survival strategy, prolonging embryo longevity and enabling survival under adverse environmental conditions such as extended drought and extremes of temperature. Seed longevity is a complex trait determined by the interaction of multiple genetic and environmental factors and can vary even between closely related ecotypes [1,2]. Most plant species produce seeds that can withstand drying to low moisture content on the mother plant and harsh environmental stresses such as freezing, termed ‘orthodox’ seeds. In contrast, seeds that retain higher hydration levels at maturity and are unable to withstand desiccation and freezing storage conditions are termed ‘recalcitrant’, although some seeds display gradients of desiccation and freezing sensitivity [3]. Plant species that produce desiccation tolerant seeds predominate in temperate latitudes and represent the majority of crop plants, whereas recalcitrant seeds are more commonly found in tropical latitudes [4]. Survival in the dry state is termed anhydrobiosis and leads to metabolic quiescence, greatly extending the seed lifespan [5]. Orthodox seed longevity varies enormously between species, with the maintenance of seed viability extending from years to millennia [6]. For example, 2000-year-old date palm seeds originating from the ancient site of King Herod’s Palace near Jerusalem were capable of germination and produced viable trees [7]. A specialised developmental programme prepares cells in the embryo for tolerance of extreme dehydration in orthodox seeds (Figure 1). However, the cycle of desiccation, quiescence and rehydration (imbibition) is nevertheless associated with high levels of cellular damage [3]. This review will highlight recent progress in our understanding of the factors that minimise damage and promote cellular repair in germinating orthodox seeds, with a particular focus on the roles of genome maintenance mechanisms.

## Desiccation tolerance and longevity

Desiccation tolerance is established during seed maturation on the mother plant through the programmed expression of cellular factors that protect against stresses associated with dehydration and rehydration. Seed maturation is promoted by the plant hormone abscisic acid (ABA) and is under the control of LAFL transcription factors (**L**EC2, **A**BI3, **F**US3 and **L**EC1) [8]. Production of late embryogenesis abundant (LEA) proteins and sugars in the final phase of seed development protects cytoplasmic components in the dry seeds and confers desiccation tolerance [9]. Later stages of seed maturation are important for increasing seed longevity, associated with the expression of genes involved in RNA processing, translation and defence [10,11]. The drying phase of seed maturation is characterised by reduction of seed water content to 5–15% fresh weight. The accumulation of sugar and protective proteins provides dry matter in the cytosol which limits cellular shrinkage and therefore may reduce viability loss [3]. The removal of free water leads to a phase transition as the cytoplasm reduces mobility from a fluid to glassy state [12]. This results in metabolic quiescence and increased longevity [13]. Seeds can retain desiccation tolerance over multiple cycles of hydration and desiccation but irreversibly lose desiccation tolerance as germination progresses [14]. Desiccation tolerance can be re-introduced by treatment with ABA or PEG [15,16], which has been used in transcriptomic and proteomic studies to identify factors conferring resistance to dehydration stress. For example, the re-establishment of desiccation tolerance in pea was accompanied by increased levels of stress responsive proteins including peroxidases and glutaredoxins [17]. In *Medicago truncatula*, re-introduction of desiccation tolerance coincided with increased levels of *ABA INSENSITIVE 5* (*ABI5*) transcripts, whereas *Mtabi5-1* and *-2* mutant lines remained desiccation sensitive [18]. Re-acquisition of desiccation tolerance in *M. truncatula* was preceded by a transcriptional programme with similarities to seed maturation and repression of metabolism and cell cycle activity [19]. Changes in gene expression associated with the re-acquisition of desiccation tolerance were reflected in chromatin accessibility and histone modifications [20]. Protein levels of cell cycle and glycolysis enzymes (e.g. phosphoglycerate kinase) were reduced as desiccation tolerance was re-established, as shown in seeds of *Caragana korshinskii* Kom [21]. Cellular events during maturation are important for seed survival and disruption of these processes, either through genetic mutations or premature harvesting, can severely reduce seed longevity. In extreme cases, for example, *abi3* mutations, seed survival is dramatically reduced [22,23]. The presence of photosynthetic components in seeds may contribute to the poor longevity of early harvested seeds [24]. Defects in the formation of the protective seed coat also significantly reduce long-term seed survival, which may result from the increased permeability allowing greater access of water and moisture [25]. Genetic screens for seed longevity, including Quantitative Trait Loci (QTL) mapping, resulted in the identification of factors associated with seed maturation in a range of species. For example, in Arabidopsis, biosynthesis genes for the oligosaccharides galactinol and raffinose were identified as determinants of longevity [26].

## Seed germination

Germination is initiated by water uptake (imbibition), resulting in activation of cellular metabolism, and is completed with the emergence of the young root (radicle) through the seed coat (testa) [4,27,28]. The embryonic plant is reliant on the storage reserves laid down during seed maturation to support germination and early seedling growth [4]. Once the seedling has established a root system, it can acquire nutrients from the soil. Emergence of the shoot from the soil provides access to light for photosynthesis. Seed germination and seedling establishment are particularly vulnerable stages of the plant life cycle [29]. During this developmental transition, plants are highly susceptible to environmental stresses. High-vigour seeds display rapid, synchronous germination tolerant of environmental stresses and establish robust seedlings [29]. Decreasing seed vigour is manifest as a decline in the speed and uniformity of germination, in which a progressively extending lag phase to the completion of germination (radicle emergence) finally culminates in viability loss. Seed ageing slows germination and weakens subsequent seedling growth, significantly increasing mortality rates [30]. The rate of seed ageing is accelerated under storage conditions of elevated temperature and relative humidity and is also dependent on harvest quality and genetic factors [1,31]; seed ageing has been the subject of a number of excellent recent reviews [3,13,24,30–33].

## The biochemistry of seed ageing

**Figure 2:**
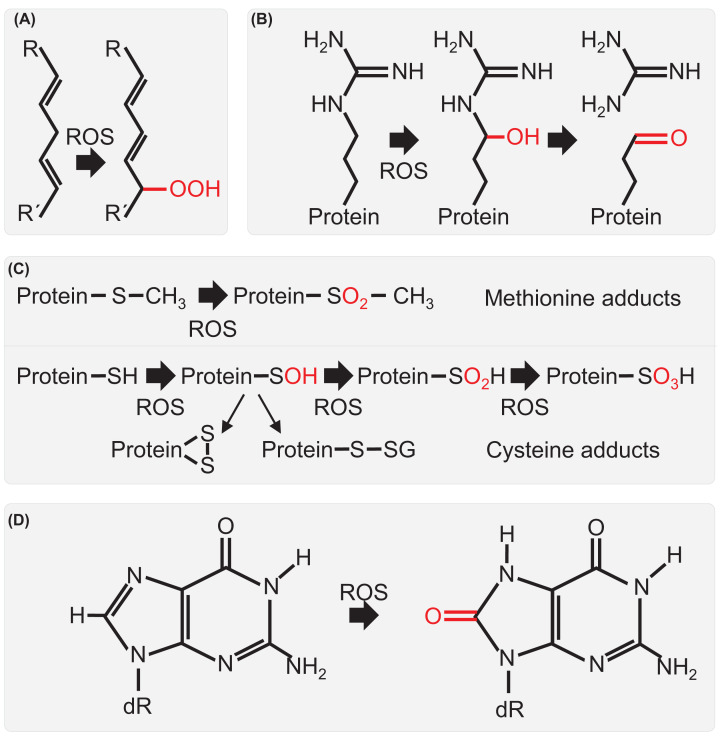
Oxidation products in seeds Examples of cellular macromolecular adducts produced by reactive oxygen species. (**A**) Lipids are oxidised to form lipid peroxides and lipid hydroperoxides (A) directly by reactive oxygen species (ROS) or through reactions with other metabolites [153]. (**B**) Amino acid side chains (e.g. arginine) are oxidised to form carbonyl groups on proteins [154] (**C**) Oxidation of methionine results in production of methionine sulfone. Progressive oxidation of cysteine forms sulfenic, sulfinic and sulfonic acids. Sulfenic acid can undergo further reactions to form disulphide bonds and intermolecular disulphide bonds with glutathione (gluthionylation) and other proteins [59]. (**D**) Oxidation of base guanine to form 8-oxoguanine (8-oxoG) is the major oxidative damage product in DNA (8-oxo-2′-deoxyguanosine) [155] and a similar product (8-hydroxyguanosine [8-OHG]) is a prevalent result of RNA oxidation [156].

Biochemical analysis reveals the cellular damage associated with loss of germination vigour and reduced seed viability. Seed ageing results in the accumulation of oxidation products of proteins, DNA and lipids as the cellular environment becomes increasingly oxidised (Figure 2) [3,33]. Upon seed rehydration, termed imbibition, the influx of water further exacerbates cellular damage, in part arising from the loss of compartmentation as membranes become leaky, exacerbating the damaging effects of ageing [30]. Thus, the protective factors synthesised during seed maturation confer desiccation tolerance but are not sufficient to prevent the accumulation of cellular damage over time, resulting in seed ageing, compromising germination and eventually culminating in loss of seed viability. Due to the long timescale of seed deterioration during storage under optimal conditions in many species, protocols of accelerated ageing are widely utilised to simulate the natural ageing process [34]. Although natural and accelerated ageing share some similarities, differences in cytoplasmic molecular mobility and biochemical reactions in conditions of high relative humidity also result in mechanistic differences between seed ageing under dry and humid conditions [24,32]. As such, accelerated ageing may not be the ideal model for studying seed deterioration under controlled conditions in seed banks, but may reflect some natural environmental conditions better than dry ageing [32]. The following sections examine some of the major cellular changes which occur in ageing of orthodox seeds, with all studies using accelerated ageing unless otherwise stated.

## Redox changes in the dry seed

The cellular redox state is governed by the antioxidant glutathione, which exists in reduced (GSH) and oxidised forms (GSSG) [35]. GSH is the most abundant water-soluble antioxidant in orthodox seeds [36]. Seed ageing across a wide range of species and ageing regimes results in an elevated GSSG/2GSH ratio, indicative of increasingly oxidising values as seed lots lose viability [36–40]. This link between ageing and redox state is supported by the observation that Arabidopsis ecotypes with higher levels of glutathione display increased seed longevity [41]. The importance of redox homeostasis is supported by a Genome-Wide Association Study (GWAS) of 270 Arabidopsis ecotypes that identified several genes linked to increased longevity, including *DEHYDROASCORBATE REDUCTASE 1* (*DHAR1*) [42]. Analysis of *dhar1* mutant lines confirmed roles in promoting resistance to seed ageing, with mutants displaying ∼60% viability after a year of natural ageing, compared with wild-type seed viability of ∼95%. Arabidopsis seeds deficient in FUMARYLACETOACETATE HYDROLASE (FAHD1A) resulted in increased levels of antioxidants (ascorbic acid and dehydroascorbate) and more reducing cellular conditions (a lower GSSH/2GSH ratio). This was indicative of altered redox metabolism during seed maturation in the absence of FAHD1A. The *fadh1a* mutant lines displayed reduced thermodormancy and increased resistance to seed ageing at 60–75% relative humidity, consistent with the more reducing cellular redox state delaying seed ageing [43]. ROS signalling plays important roles in cellular physiology and dormancy alleviation in seeds, but high levels cause extensive cellular damage, seed ageing and loss of viability [44]. Oxidative damage arises from the increased cytoplasmic mobility during seed ageing at elevated humidity and temperature damage as the cytoplasm transitions from an intracellular glass to a fluid state [3,45,46]. Seeds stored under highly controlled, low humidity environmental conditions display much slower rates of ageing and a different spectrum of damage products compared to seeds subjected to rapid ageing regimes involving warm, humid conditions [38]. Under conditions of reduced cytoplasmic mobility (RH 11–30%), oxidation of cellular components and seed ageing was dependent on the availability of ambient oxygen [47]. In contrast, high humidity (60–80% RH) led to seed ageing regardless of O_2_ availability. Under these conditions of high cytoplasmic mobility, ageing was associated with loss of glutathione rather than cellular oxidation [47]. Differences between slow ageing in drier conditions and accelerated ageing of seeds with higher water content may reflect increased enzyme activity in seeds exposed to high humidity [38]. In the natural environment, seeds are likely to experience a range of fluctuating temperature, humidity and hydration states, all of which will influence the nature of cellular stresses that result from ‘dry’ and ‘wet’ ageing [13]. While the accumulation of specific cellular damage products may differ depending on the environmental conditions seeds encounter, a unifying feature is the increased oxidation of the cells in ageing seeds [37].

## Membrane damage

Lipid oxidation (Figure 2A) leads to membrane damage, loss of structural integrity and cellular solute leakage from membranes. The correlation between lipid peroxidation and seed ageing was the subject of conflicting reports in a number of pre-genomic era studies, although the differences in results were potentially attributable to the seed ageing conditions utilised in the different labs [48,49]. Recently, both lipid oxidation and hydrolysis were shown to correlate with loss of seed viability in ageing of dry stored wheat seed, linking ROS to loss of cellular integrity and lipid peroxidation [50]. Antioxidants play important roles in minimising cellular damage in ageing seed. Arabidopsis mutants deficient in tocopherol (vitamin E) synthesis, a lipophilic antioxidant that combats lipid peroxidation, are hypersensitive to accelerated ageing [51]. Moreover, mutant seedlings display defects such as abnormal cotyledon expansion and white patches on cotyledons, consistent with lipid peroxidation damage. Damage to lipids compromises membrane integrity, which, together with cell death, results in solute leakage from aged seeds. Conductivity tests of solutes leaked from ageing seed lots provide good predictions of seed viability [30,52].

## Protein modification

Oxidation and carbonylation are principal modifications which impair protein function in the ageing seed (Figure 2B) [53,54]. Seed ageing correlates with significantly increased levels of irreversible protein carbonylation, which can impact on protein function [34]. Abundant seed storage proteins and metabolic enzymes are the principal targets of these modifications in Arabidopsis seeds [53]. Cruciferin storage proteins in the Arabidopsis seed form important targets for oxidative modification by ROS, potentially minimising oxidative damage to the seed. Cruciferin deficient mutants are significantly more sensitive to oxidative stress [55]. Amino acid side chains and the peptide backbone are also subject to oxidation [56]. Cysteine and methionine are particularly sensitive to even mild oxidative stress and these forms of damage can be repaired. Protein oxidation can lead to formation of methionine sulfoxide residues and reversal of this damage is catalysed by METHIONINE SULFOXIDE REDUCTASE (MSR) [57]. MSR levels correlate with seed lifespan in varieties of *Medicago truncatula*. The conversion of aspartate residues to isoaspartyl residues is associated with ageing and causes protein mis-folding that can be reversed by L-ISOASPARTYL METHYLTRANSFERASE 1 enzymes. These were identified as important factors which confer of Arabidopsis seed longevity and vigour and are found at particularly high levels in sacred lotus seeds, which exhibit extreme longevity [58]. Oxidation of cysteine produces sulfenic, sulfinic and sulfonic acidic derivatives, and sulfenic acid can undergo further reactions to produce disulphide bonds within or between proteins, or with glutathione (glutathionylation) (Figure 2C) [59].

## RNA modification

Translation plays a critical role in germination, but cellular RNA is particularly sensitive to oxidative damage [60,61]. Loss of both ribosomal RNA and messenger RNA integrity has been linked with seed ageing, representing a sensitive predictor of seed ageing in dry storage for a number of studies [62–65]. Changes in mRNA levels have been detected in dry seeds in both natural and accelerated ageing and are associated with dry-after-ripening [66,67]. Oxidative damage to mRNA can cause the fragmentation observed in soybean seeds over 20 years storage [68]. This damage appeared random, affecting longer transcripts more than shorter ones, and consistent with gradual non-enzymatic degradation of transcripts over time, although some degraded transcripts were present even in new seed lots. Similar conclusions were drawn from a study in Arabidopsis, leading to an estimate of mRNA damage in the dry seed at a rate of ∼1 × 10^−4^ per nucleotide per day, equating to each nucleotide in a transcript suffering damage once every 30 years [69]. In rice, natural ageing and accelerated ageing resulted in similar transcriptional changes in the dry seed, with mRNA degradation occurring at higher rates in a subset of transcripts [70]. These results are consistent with previous reports showing targeted RNA degradation in desiccated seeds [67].

## DNA damage and repair

DNA represents the genetic material of inheritance and the template for both gene expression and DNA replication. However, DNA is inherently unstable in the aqueous, cellular environment. The constant accumulation of damage products can result in delayed growth, mutagenesis or cell death if unrepaired [71]. Desiccation greatly reduces the rate of DNA damage, but also prevents repair processes. As a result genome damage can accumulate over extended periods of storage and exposure to elevated humidity, with additional genome damage incurred during rehydration [72]. DNA damage is increased by environmental stresses such as UV or the endogenous by-products of metabolism, in particular ROS [73]. Base damage is the major DNA lesion and predominantly results in oxidation of guanine to form 8-oxoguanine (8-oxoG, Figure 2D) which is removed during repair to form an abasic site. The dry, quiescent maize embryo accumulated several million abasic sites per cell after two years of natural ageing, increasing four-fold on imbibition [74]. DNA double strand breaks (DSBs), representing a broken chromosome, are a highly cytotoxic form of DNA damage. Across the kingdoms of life, anhydrobiosis is associated with the accumulation of DSBs. For example, desiccation of the desert dwelling bacterium *Deinococcus radiodurans* results in cumulative fragmentation of the genome into hundreds of small pieces which is then rebuilt on rehydration [75]. Similarly, tardigrade invertebrates accumulate genome damage in the dry state [76]. In orthodox seeds, genome stress is evident as extensive chromosome fragmentation observed even in high quality, unaged seeds resulting in high levels of chromosomal abnormalities relative to other stages of plant development [72,77]. Levels of chromosomal breaks are significantly increased by adverse environmental conditions encountered in seed development, storage and imbibition [78]. The accumulated genome damage in ageing seeds results in elevated frequencies of cytogenetic abnormalities, including anaphase bridges produced from chromosomal fusions [72,77].

**Figure 3:**
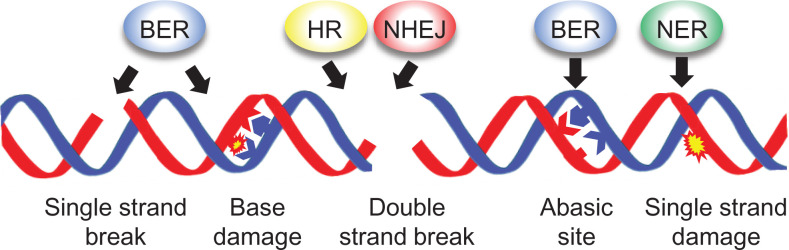
DNA damage and repair activities in seeds DNA damage results in single and double stranded DNA breaks, base loss and damage to damage to the sugar-phosphate backbone. This requires the activities of the major DNA repair pathways, all of which influence germination. BER: Base Excision Repair; NER: Nucleotide Excision Repair; NHEJ: Non-Homologous End Joining; HR: Homologous recombination. Alternative end-joining (alt-EJ) pathways operate in plants, including DNA polymerase theta (POLQ) mediated end-joining (TMEJ) [157], although functions in seeds are not well characterised. However, recently a *ku70 polq* double mutant was reported to have reduced germination [158].

Eukaryotic cells have evolved powerful and complex repair and response mechanisms to minimise the threat to cellular survival and safeguard the fidelity of genetic information. Although many DNA repair pathways are conserved in eukaryotes, plants display key differences in genome maintenance mechanisms, reflecting specific requirements in their sessile, autotrophic lifestyle [73]. Safeguarding the genetic integrity of meristem cells is particularly important as they are the progenitors for plant development [79]. Seeds display activity of the major pathways for repair of DNA damage, including base and nucleotide excision repair (BER, NER) and the repair of chromosomal breaks by non-homologous end joining (NHEJ) and homologous recombination (HR) (Figure 3). These DNA repair activities promote seed vigour and viability [85].

Base Excision Repair (BER) is initiated by the detection and removal of specific damaged bases by DNA glycosylases, for example, 8-OXOGUANINE GLYCOSYLASE (OGG1) which removes 8-oxoG (Figures 2D and 3) [80]. The expression of *OGG1* DNA glycosylases is increased during seed imbibition [81,82]. Overexpression of *OGG1* decreased 8-oxoG levels in seeds and conferred resistance to controlled deterioration in addition to a range of abiotic stresses such as heat in germination [80,83]. Conversely, seed lacking endonuclease activity required for BER, termed APURINIC ENDONUCLEASE-REDOX PROTEIN (ARP), exhibited hypersensitivity to seed ageing [83]. Nucleotide Excision Repair (NER) plays a key role in the removal of bulky DNA lesions and NER genes are expressed during seed development [84]. Functional roles for NER in seeds are revealed by analysis of mutants lacking the NER factor XERODERMA PIGMENTOSUM B (XPB1) which display reduced germination [85]. DSBs are a highly mutagenic and cytotoxic form of DNA damage which are repaired by NHEJ and HR. In somatic plant cells, NHEJ activities predominate, although HR is important during DNA replication (S-phase) and is upregulated in plant meristem cells [86]. NHEJ involves direct joining of broken DNA ends without the requirement for a template, resulting in random-end-joining. In contrast, HR uses a homologous DNA template to accurately restore the broken chromosomes through homology-mediated repair [73]. Arabidopsis mutants deficient in HR factors displayed hypersensitivity to seed ageing [87]. Germination of irradiated maize *rad51* mutant seed was delayed relative to wild type, consistent with an increased requirement for HR as seeds lose vigour [88]. Similarly, mutation in NHEJ pathway factors DNA LIGASE 4 (LIG4), DNA LIGASE 6 (LIG6) and KU80 results in hypersensitivity to accelerated ageing, indicating essential functions in maintaining genome integrity in germination [78,87]. Arabidopsis *lig6 lig4* double mutants also displayed hypersensitivity to natural seed ageing for 10 years under ambient conditions [89]. The naturally aged DNA ligase deficient lines displayed significantly elevated frequencies of programmed cell death (PCD) in the apical meristem of roots three days post-germination. This indicates that DNA repair activities are required for recovery from seed ageing under both natural (long-term, dry ageing) storage conditions and after accelerated ageing at elevated temperature and humidity [78,89].

## Cellular responses to seed ageing

The stresses associated with seed ageing result in transcriptional responses, as revealed by a number of microarray and RNA-seq studies [33]. Accelerated ageing results in changes to transcript levels in the dry seed, suggestive that high humidity can increase cellular hydration to levels that support transcription, at least in some cells. Changes in transcript levels include components of the translation machinery, as observed in pea seeds subjected to ageing treatments [90]. Imbibition of aged Arabidopsis seeds leads to large scale transcriptional changes that significantly differ to unaged, high quality seeds [91]. Imbibed aged Arabidopsis seeds displayed stress responses associated with heat shock and increased expression of genes involved in RNA metabolism [91]. Consistent with these findings, long-lived Arabidopsis ecotypes display elevated transcript levels of heat shock factors and RNA processing genes, with the corresponding mutant lines displaying altered sensitivity to ageing [41]. An earlier study reported increased expression of *Glutathione S Transferase U22* in dry aged seeds, potentially resulting from increased oxidative stress [92]. Rice mutants with reduced anti-oxidant levels displayed reduced seed longevity. These plants exhibited increased expression of an E3 ubiquitin ligase of ARABIDOPSIS TOXICOS EN LEVADURA family and the Arabidopsis orthologue, ATL5, was shown to be required for seed longevity, potentially acting as a regulator of transcription [93]. Changes in protein phosphorylation have been reported in imbibing seeds [94], although the effects of ageing on post-translational modifications are less well characterised, other than oxidative products that are abundant in aged seeds [34,95]. The transcriptional DNA damage response, comprising hundreds of genes, is induced early in imbibition of Arabidopsis and barley seeds. This reflects the requirement for DNA damage responses in germination to repair striking levels of genome damage sustained during desiccation, quiescence and rehydration, even in high quality, unaged seed [78].

## Mitigating the effects of desiccation and quiescence on seed genome stability

### Chromatin dynamics and epigenetic changes in seeds

DNA repair, DNA replication and transcription all take place in the context of chromatin, with DNA packaged by histones into nucleosomes and higher order structures. Phosphorylation of HISTONE H2AX is a conserved response to DNA damage in eukaryotes and loss of H2AX in Arabidopsis seeds resulted in hypersensitivity to accelerating ageing [96] Maturation of *Phaseolus vulgaris* seeds is accompanied by elevated expression of transcripts associated with chromatin structure and DNA repair [84]. Arabidopsis HISTONE H3.3 is deposited on the 5′ regulatory region of genes during seed development [97]. Mutant plants lacking H3.3 produced low viability seeds, and of the few seeds that germinated, only a small number progressed through development, with none producing seeds. The mutants displayed reduced chromatin accessibility and defects in germination associated with transcription. In some desiccation tolerant organisms, specialised genome protective proteins have been identified and chromatin may help reduce damage in the dry state [98]. Chromatin in Arabidopsis seeds remains compacted until the completion of germination and in the hydrated dormant seed [99]. Nuclear size is also reduced, but appears under distinct control to that of chromatin condensation, with roles for ABA signalling through ABI3 [99]. Factors in seeds that confer desiccation tolerance include sugars and proteins which accumulate during seed maturation and protect membranes and proteins from damage incurred during dehydration [13]. However, their role in protecting DNA in the dry state is less well defined, whereas in other desiccation tolerant organisms specialised genome protective proteins have been identified [98]. 9-CIS-EPOXYCAROTENOID DIOXYGENASE (NCED6), a key enzyme in ABA biosynthesis, is progressively silenced at the transcriptional and chromatin level during germination, potentially correlating with loss of desiccation tolerance [100]. Changes in chromatin dynamics of several genes were associated with the re-imposition of desiccation tolerance in Medicago [20]. Epigenetic modification modulates chromatin structure and compaction, thereby controlling accessibility of DNA repair, DNA replication and transcriptional machinery [101]. DNA methylation has been shown in a number of studies to change with seed ageing of both orthodox and recalcitrant species. Accelerated ageing results in altered DNA methylation in dry seeds which increased post-germination, along with levels of mutagenesis [102,103]. Interestingly, seed longevity was shown to be an adaptive response that was inherited through a generation, indicative of epigenetic changes in response to the environment [103–105]. DNA repair and damage responses are linked to dynamic changes in plant histone modification. Moreover, actively transcribed and silenced regions of the genome are subject to different rates and mechanisms of genome repair, with DNA repair complexes interacting with both chromatin remodelling and transcriptional machinery [106]. Furthermore, DNA repair mechanisms are also dependent on cell cycle stage [107]. Thus, genome repair in seeds will be determined by chromatin compactness, transcriptional activity and the progression of germination to cell cycle activation.

### Germination and cell cycle control

Resumption of cellular metabolism is initiated within minutes of seed imbibition, with cell cycle activity increasing several hours later. Genome damage accumulated in the embryo must be repaired prior to cell cycle activation in order to minimise growth inhibition and mutation of genetic information. In Arabidopsis seeds, most cells are arrested in G1, and S-phase (DNA replication) in the root apical meristem (RAM) marks activation of the cell cycle around the time of germination [108,109]. The shoot apical meristem (SAM) activates during post-germinative growth, around 12h later than the RAM in Arabidopsis [108]. Nuclei in cell in G1 phase undergo a transient increase in oxidation as part of the cell cycle [110]. However, Arabidopsis mutant lines with reduced ascorbate experienced higher levels of oxidative stress in the embryonic root and delayed cell cycle progression [110].

**Figure 4:**
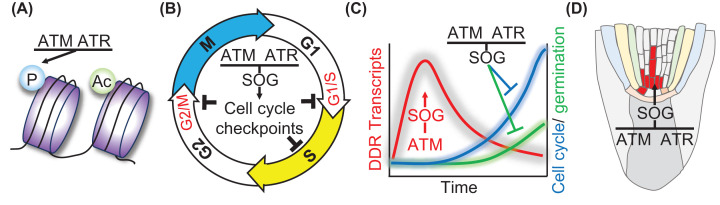
DNA damage responses in plants The DNA damage signalling kinases ATAXIA TELANGIECTASIA MUTATED (ATM) and ATM AND RAD3-RELATED (ATR) orchestrate plant cellular responses to DNA damage, with major roles played by the transcription factor suppressor of γ1 (SOG1). (**A**) Post-translational modification of proteins includes acetylation (Ac) of histones and phosphorylation (P) of hundreds of proteins including the DNA damage signalling factors HISTONE H2AX (H2AX) and SOG1 [96,159–161]. (**B**) DNA damage results in arrest of the cell cycle at the transitions between G1- and S-phase, G2- and M-phase and within S-phase (intra-S) [112]. (**C**) The DNA damage response (DDR) in seeds results in the transcriptional regulation of hundreds of genes in the first few hours of imbibition and delays both DNA replication and germination [113,162]. (**D**) DNA damage can lead to the switch from the mitotic cell cycle to endocycles or programmed cell death in meristem cells, revealed by propidium iodide staining of non-viable stem cell initials (coloured red) [115,116].

The cytotoxic effects of accumulated genome damage in plants are mitigated by the activation of response mechanisms [111,112]. In plants, DDR activation is orchestrated by the protein kinases ATAXIA TELANGIECTASIA MUTATED (ATM) and ATM AND RAD3-RELATED (ATR) [113], with many responses acting through the transcription factor SUPPRSSOR OF GAMMA 1 (SOG1) [114]. SOG1 is unique to plants but is considered functionally analogous to p53 in mammalian cells. ATM is activated by DSBs whereas ATR is activated by single stranded regions of DNA originating in DNA replication or DSB processing ATR, but both kinases act through SOG1. Downstream responses of the plant DDR include the transcriptional DNA damage response, activation of DNA repair factors, PCD and activation of cell cycle checkpoints or a switch to endocycles that together maintain genome integrity and minimise formation of mutations (Figure 4) [113,115,116]. In plants, PCD of cells with compromised genomes in meristematic tissues represents an effective mechanism to maintain meristem function [115]. DNA laddering characteristic of plant cell death was reported in pea and sunflower, coincident with loss of seed viability [90,117]. Cell cycle checkpoints restrict growth in the presence of damage that would otherwise result in severe genome instability, meristem failure and death [118]. In Arabidopsis seeds, checkpoint deficient *atm* and *atr* mutants display apparently increased seed viability relative to wild-type after ageing. However, seedlings germinated from aged mutant seeds display reduced survival on soil and *atm* mutant seedlings display elevated levels of chromosomal abnormalities [77].

Regulatory proteins integrate environmental and developmental signals to control cell cycle activity, including WEE1 which controls entry to S-phase [111], and homologue of the yeast cell size (wee) mutant. In response to DNA damage, Arabidopsis ATM induces expression of the SIAMESE/SIAMESE-RELATED genes *SMR5* and *SMR7* which results in cell cycle arrest in both seeds and mature plants [77,111]. The ATM DNA damage checkpoint functions to delay germination in response to genome damage in ageing seeds, underlying the extending lag-period to germination as seed vigour declines. Thus, ATM-dependent control of germination helps mitigate the effects of genome damage in low vigour seeds by integrating germination progression with genome surveillance and activation of DNA damage response [77]. These cell cycle checkpoint activities function to preserve genome stability and mitigate the growth-inhibitory effects of damage accumulated in dry seeds.

### Seed ageing and seedling establishment

Germination is defined as the emergence of the young root through the seed coat (testa) [4]. During the subsequent phase of growth, the emergent seedling is dependent on the mobilised nutrient storage reserves contained within the seed until the root and shoot systems are capable of mediating autotrophic growth [4]. Seedling establishment is a critical phase in the plant life cycle which is highly susceptible to adverse environmental conditions [27]. Successful establishment is required for optimal crop yields and is dependent on high seed vigour [29]. Rapid, synchronous germination supports seedling establishment that is tolerant of adverse environmental conditions [119–121]. The emerging seedling requires rapid development of root and shoot systems to enable the transition to autotrophic growth. Delayed root growth, for example, restricts the ability of the germinating seed to access water required to drive cell expansion and early seedling growth [29]. Mechanical soil impedance to seedling emergence restricts both root and shoot elongation and is highly dependent on soil hydration and physical composition. Water-logged or dry soils require high growth vigour to promote seedling emergence [29]. Low vigour, weaker seedlings display increased mortality and greater susceptibility to biotic and abiotic stresses including fungal pathogens, insects and physical stresses imposed by the surrounding soil [122]. The factors that lead to poor seedling growth after seed ageing remain obscure at the molecular level. However, low vigour seeds germinate to produce seedlings with high levels of genome instabilty, resulting in extensive chromosomal abnormalies and increased intra-chromosomal recombination [72,77,89].

Recent work showed that imbibed Arabidopsis seeds exhibit high resistance to DNA damage (X-irradiation) in contrast with seedlings. This resistance is lost as seeds progress to germination, coinciding with increasing cell cycle activity [87]. Seeds minimize the impact of genome damage observed at later stages of plant growth by reducing meristem disruption and delaying SOG1-dependent programmed cell death in response to genotoxic stress [87]. SOG1 activation of cell death in the RAM is delayed several days post-germination in response to both X-irradiation and natural seed ageing [87,89]. Thus, seeds promote post-germinative root growth to enable rapid seedling establishment and transition to independent resource acquisition and autotrophy. The distinct cellular responses of seeds and seedlings to genome damage may be attributed to low cell cycle activity in early-imbibed seeds, reflected in distinct transcriptional DNA damage response observed in plants at these different stages of development [87]. Seedlings germinated from aged mutant seed deficient in the DNA-damage cell cycle checkpoints factor SOG1 establish poorly on soil, although the seeds display apparent resistance to ageing, as observed for *atm* and *atr* mutants [77,87]. Thus, low cell cycle activity, together with cell cycle checkpoints and powerful DNA repair activities, function in germination to promote successful seedling and early growth.

The mutagenic potential of DNA damage accumulated in seeds on subsequent plant growth remains largely unknown. Analysis of genome instability in seedlings germinated from ageing Arabidopsis seeds identified striking increases in both frameshift mutations (using a microsatellite stability reporter line) and genome stability (using an intrachromosomal recombination reporter) as germination vigour declined [89]. Thus, elevated levels of genome damage incurred in the seed stage of the plant life cycle potentially impact on subsequent plant development. Moreover, the mutagenic effects of seed ageing has implications for the genome stability of natural plant populations under climate change given that environmental conditions in seed development influence seed quality.

### Dormancy and genome damage

Dormancy is a block to germination which prevents germination under conditions where non-dormant seeds germinate. Dormancy is released over time or after specific environmental dormancy-breaking signals are received (e.g. cold and light) [123]. The correct decision for when a seed germinates, in terms of season and local environment, is critical to plant survival and natural ecosystems. The preservation and dry storage of crop seeds in agriculture contrasts with the natural environment in which seeds persist in the soil seed bank, periodically undergoing wet and dry cycles or prolonged periods of hydration and desiccation dependent on climate [29]. Seeds integrate multiple inputs from genetic and environmental sources that optimise germination for an individual seed and disperse the progeny of the mother plant over time [123]. In the seed soil bank, seeds may go through several cycles of hydration and dehydration in the dormant state, retaining desiccation tolerance, and only germinate following re-imbibition when non-dormant [124]. Hydrated dormant seeds are metabolically active but do not initiate DNA replication, unlike non-dormant counterparts [125]. The dormant state and retention of desiccation tolerance may therefore be associated with suppression of cell cycle activation/progression. Notably, seeds that are maintained in a hydrated state during maturation show reduced genome damage and chromosomal defects [126]. Furthermore, seeds undergoing wet-dry cycles in the soil seed bank display seasonal fluctuations in genome surveillance and DNA repair transcripts, including ATM and ATR [77] which correlate with changes in dormancy and germination potential in response to environmental signals including temperature and soil moisture content [77]. However, to date genome maintenance in dormancy has not been further investigated at the molecular level.

### Seed priming and genome repair

Seed germination and establishment in many commercial species are improved by pre-germinative priming treatments in which controlled hydration facilitates cellular repair processes [127–129]. Primed seeds are then re-dried before completion of germination and loss of desiccation tolerance. Seedling establishment for many commercial species, typically >70% in the case of sugar beet, can be increased ∼10% by vigour enhancement through seed priming [129]. The improved growth vigour of primed seeds also confers resistance to biotic and abiotic stresses encountered in the field, resulting in significant and sustainable yield increases [29]. However, the molecular basis for the improvement of germination vigour conferred by seed priming is not fully understood, although resumption of metabolism is likely to facilitate cellular repair processes [28,130]. DNA synthesis, but not cell division, is detectable during priming of leek seeds (*Allium porrum*), and primed *Brassica oleracea* seeds germinate faster than unprimed controls, displaying very high rates of DNA synthesis associated with rapid cell division promoting early seedling growth [131,132]. Priming results in large scale changes in transcript and protein levels as pre-germinative metabolism progresses, including expression of DNA repair factors and increased activity of the protein repair enzyme L-ISOASPARTYL METHYLTRANSFERASE [133–137]. Chromosomal defects were reduced in primed seeds, coincident with increased ‘normal’ germination (lower incidence of seedlings with developmental abnormalities as defined by the International Seed Testing Organisation [30]) [138]. Collectively, these results support the role of priming in germination advancement through pre-germinative repair processes [139]. An element of the germination vigour conferred by priming may also result from stresses incurred during the priming process. For example, tomato seed priming was improved by heat shock, which also led to elevated heat shock factor gene expression [140]. Priming alters ROS levels, with reduction in hydrogen peroxide accumulated in aged seeds, accompanied by increased catalase activity as seeds recover from the loss of catalase protein during ageing [135,137,141]. In wheat, priming with hot steam resulted in advanced germination, with more a more rapid shift to reducing conditions that promote progression of germination [142]. However, over-priming results in elevated ROS levels and increased genome damage [143]. Seed priming can both reduce seed longevity and change the genetic requirements for longevity in comparison to un-primed seeds [128]. Brassinosteroid (BR) signalling was implicated in the reduced longevity of primed seeds [144]. This may reflect roles of BR signalling in promoting germination, and thus mutants in BR signalling display decreased progression of germination in priming, retaining desiccation tolerance [128]. Significantly, longevity of primed seeds could be increased through the use of cell cycle inhibitors that blocked DNA synthesis [145] and the extent to which primed seeds progress through pre-germinative processes is a critical determinant of the lifespan of primed seeds in storage [128].

## Conclusions and outlook

Seed longevity is dependent on a complex interaction of genetic and environmental factors [13]. Our understanding of seed ageing and consequences in germination has advanced considerably over recent years, with research focussed on preservation of germplasm in seed banks (long-term ageing) and stresses associated with short-term ageing representing variable environmental conditions. Seed deterioration results in multiple stresses which disrupt redox homeostasis and damage cellular components. In this review, we focussed on the effects and associated consequences of seed deterioration on nuclear genome integrity. However, much of our understanding of plant DNA repair and response factors arises from only a limited number of model plant species. Differences in the functions and importance of DNA damage response factors are now emerging in other species, with loss of gene function having species-specific effects [146]. Our understanding of seed responses to environmental stresses is critical to predict and mitigate the consequences of climate change on crop species and ecosystems [147]. The roles of seeds in future space exploration are being explored on the International Space Station, with research to investigate the effects on germination vigour and stress responses [148,149]. However, important questions remain: what mechanisms link cellular damage to control of germination, and determine survival or loss of viability? Do specific genome protection factors or mechanisms exist in seeds, as observed in other anhydrobiotic organisms? To what extent does cellular damage accumulated in the seed affect seedling performance in the field or the survival and genome stability of wild species? How will seed performance be affected by increased environmental stresses associated with changing climates in agriculture and wild populations? Applications of new technologies will help us answer these questions. As we gain more insight into how seeds integrate cellular damage with successful germination, and the longer-term effects of this damage on seedling establishment, we will be able to develop new tools and approaches to produce climate-resilient crops and enable long-term germplasm conservation for future generations.

## Open Access

Open access for this article was enabled by the participation of University of Leeds in an all-inclusive *Read & Publish* agreement with Portland Press and the Biochemical Society under a transformative agreement with JISC.

## Abbreviations

ABA: abscisic acid
ARP: APURINIC ENDONUCLEASE-REDOX PROTEIN
ATM: ATAXIA TELANGIECTASIA MUTATED
BR: brassinosteroid
LEA: late embryogenesis abundant
NHEJ: non-homologous end joining
QTL: Quantitative Trait Loci
SAM: shoot apical meristem
SOG1: SUPPRESSOR OF GAMMA1
